# Comparison of Prognosis Between Hybrid Debranching Surgery and Total Open Arch Replacement With Frozen Elephant Trunk for Type A Acute Aortic Syndrome Patients

**DOI:** 10.3389/fcvm.2021.689507

**Published:** 2021-07-27

**Authors:** Jinzhang Li, Lei Li, Maozhou Wang, Haiyang Li, Lizhong Sun, Yongmin Liu, Ruixin Fan, Zonggang Zhang, Chengwei Zou, Hongjia Zhang, Ming Gong

**Affiliations:** ^1^Department of Cardiac Surgery, Beijing Anzhen Hospital, Capital Medical University, Beijing, China; ^2^Department of Physiology and Pathophysiology, School of Basic Medical Sciences, Capital Medical University, Beijing, China; ^3^Beijing Lab for Cardiovascular Precision Medicine, Beijing, China; ^4^Department of Cardiovascular Surgery, Guangdong Provincial People's Hospital, Guangzhou, China; ^5^Department of Cardiac Surgery, People's Hospital of Xinjiang Uygur Autonomous Region, Urumqi, China; ^6^Department of Cardiovascular Surgery, Shandong Provincial Hospital Affiliated to Shandong First Medical University, Jinan, China

**Keywords:** acute aortic syndrome, frozen elephant trunk, hybrid aortic arch repair, debranching, prognosis

## Abstract

**Background:** It is unclear whether the total arch replacement (TAR) combined with frozen elephant trunk (FET) implantation and hybrid debranching surgery have a difference in the prognosis of patients with type A acute aortic syndrome (AAS). We attempted to compare the short-term and long-term prognosis of total arch replacement (TAR) combined with frozen elephant trunk (FET) implantation and hybrid debranching surgery in patients with type A acute aortic syndrome (AAS).

**Methods:** From January 2014 to September 2020, a total of 518 patients who underwent TAR with FET surgery and 31 patients who underwent hybrid surgery were included. We analyzed the post-operative mortality and morbidity of complications of the two surgical methods, and we determined 67 patients for subgroup analysis through a 1:2 propensity score match (PSM). We identified risk factors for patient mortality and post-operative neurological complications through multivariate regression analysis.

**Results:** Compared with the TAR with FET group, hybrid surgery could reduce aortic cross-clamp time, reduce intraoperative blood loss and prevent some patients from cardiopulmonary bypass. There was no significant difference in 30-day mortality between the TAR with FET group and the hybrid surgery group (10.6 vs. 9.7%). However, hybrid surgery had increased the incidence of permanent neurological complications in patients (95%CI: 4.7–35.7%, *P* = 0.001), especially post-operative cerebral infarction (*P* < 0.001). During the average follow-up period of 31.6 months, there was no significant difference in the 1-year survival rate and 3-year survival rate between the TAR with FET group and the hybrid surgery group (*P* = 0.811), but hybrid surgery increased the incidence of long-term neurological complications (*P* < 0.001). In multivariate regression analysis, surgical methods were not a risk factor for post-operative deaths, but hybrid surgery was a risk factor for post-operative neurological complications (*P* < 0.001).

**Conclusions:** Hybrid surgery is an acceptable treatment for AAS, and its post-operative mortality is similar to FET. But hybrid surgery may increase the risk of permanent neurological complications after surgery, and this risk must be carefully considered when choosing hybrid surgery.

## Introduction

Acute aortic syndrome (AAS) is a life-threatening disease. AAS includes aortic dissection (AD), intramural aortic hematoma, and penetrating atherosclerotic aortic ulcer (PAU), which have similar pathophysiological changes, clinical features, and treatment strategies (1). Stanford type-A AAS usually requires emergency treatment, especially when the lesion involves the aortic arch. If not treated, the mortality of acute type A AD can reach 50% within 48 h (2), and surgical treatment has obvious advantages over conservative treatment (3).

The treatment of type-A AAS involving the aortic arch usually includes open thoracic aortic surgery and hybrid surgery. Among open thoracic aortic surgeries, total arch replacement (TAR) combined with frozen elephant trunk (FET) implantation is a surgical method with good therapeutic effects (4), but it usually requires the use of prosthetic graft to replace the aortic arch, which is a complicated surgical technique and requires hypothermic circulatory arrest (HCA). Hybrid surgery is a new option for type-A AAS patients involving the aortic arch (5, 6). It reduces surgical trauma and does not require HCA. Hybrid surgery is a treatment method that combines surgery with interventional therapy. In interventional therapy, thoracic endovascular aortic repair (TEVAR) is a good strategy for the treatment of type-B AAS (7). However, when the lesion involves the ascending aorta and the aortic arch, TEVAR cannot be used because it affects the blood supply to the branches of the aortic arch (8). Hybrid debranching surgery can transfer the branch vessels of the aortic arch to the normal part of the ascending aorta or prosthetic graft to expand the proximal anchoring area, and then complete aortic repair through TEVAR (9). However, the safety and effectiveness of this treatment method are still uncertain (10). As the effectiveness and safety of surgical treatment have been widely recognized, surgical treatment is still the first choice for AAS involving the ascending aorta and aortic arch (2). It is necessary to analyze the prognosis of hybrid debranching surgery and open thoracic aortic surgery to analyze its safety and effectiveness.

It is not yet clear whether there is a difference between TAR with FET and hybrid debranching surgery in the prognosis of type-A AAS patients. The main purpose of this study is to compare the short-term prognosis and the long-term prognosis differences between TAR with FET and hybrid debranching surgery for type-A AAS patients with lesions involving the aortic arch, so as to provide evidence for surgeons in the selection of surgical options.

## Materials and Methods

### Participants

From the aortic disease database jointly maintained by nine medical centers in China, data on a total of 549 Stanford type-A AAS patients who underwent hybrid surgery or TAR with FET between January 1, 2014 and September 30, 2020 were collected. The ethics committee of Beijing Anzhen Hospital approved this multicenter retrospective cohort study. Patients' written informed consent was dropped due to the retrospective nature of the study. We collected demographics, surgical information, and perioperative clinical data. All patients were diagnosed as Stanford Type A AAS by experienced imaging specialists and cardiovascular surgeons through aortic computed tomography angiography (CTA), and all patients were judged by the aortic surgery team to have indications for aortic repair. Patients with missing surgical data and previous TEVAR were excluded from the study. The surgery was performed by the surgical team of the medical center at the time of the patient's admission. Surgeons were more inclined to choose hybrid surgery for older patients, but the operation method still depended on the preference of the surgeon and the requirements of the patient.

### Total Arch Replacement Combined With Frozen Elephant Trunk Implantation

**Figure 1:**
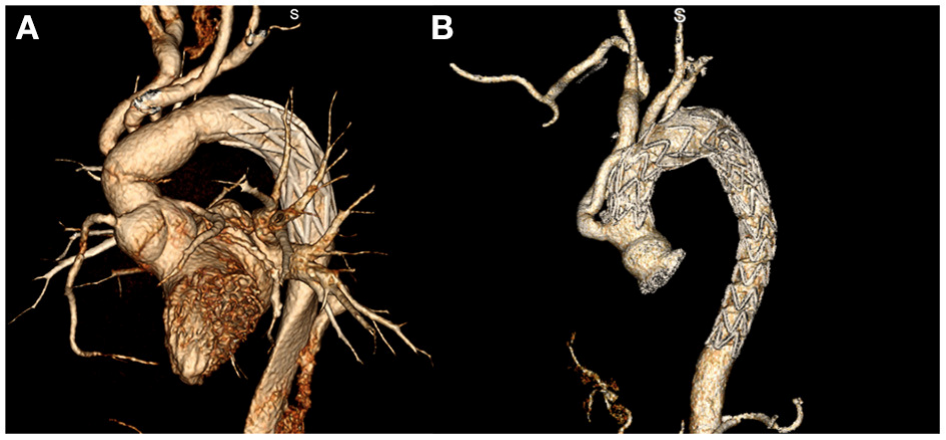
Post-operative aortic CTA reconstruction images of patients with acute aortic syndrome treated with total arch replacement combined with frozen elephant trunk implantation **(A)** and hybrid debranching surgery **(B)**.

We have previously described the process of TAR with FET in detail (4). In short, all patients received intravenous anesthesia and tracheal intubation. The venous cannula was inserted into the right atrium, and the arterial cannulas were inserted into the right axillary artery and right femoral artery to establish cardiopulmonary bypass. The aortic root repair method was determined according to the extent of the lesion, and the aortic valve replacement and ascending aortic replacement surgery were performed first. In addition, the decision to perform coronary artery bypass graft was based on whether the disease involved the coronary arteries. When the nasopharyngeal temperature dropped below 28°C, the circulatory arrest began. The selective cerebral perfusion was performed through the right axillary artery. The stent graft was placed in the descending aorta under direct vision. The four-branch prosthetic graft was used to replace the total aortic arch, and the circulation was restored after the distal end of the four-branch prosthetic graft was anastomosed. Subsequently, the left common carotid artery, innominate artery, and left subclavian artery were reconstructed in sequence, and the body temperature was gradually restored and the cardiopulmonary bypass was terminated (Figure 1A).

### Hybrid Surgery

The entire hybrid surgery was performed in the hybrid operating room equipped with a floor mounted angiography C-arm system. All patients underwent intravenous anesthesia, tracheal intubation, and median sternotomy. The open repair surgery part of the hybrid surgery was completed first. Whether to repair the ascending aorta was determined according to the extent of the lesion. For patients with lesions involving the ascending aorta, the right axillary artery and femoral artery were selected for arterial cannulation, and the right atrium was selected for venous cannulation, and then cardiopulmonary bypass was established. The right axillary artery was used to supply blood to the brain during innominate artery surgery for brain protection. After the ascending aorta was cross clamped, the ascending aorta was cut longitudinally, and cold blood cardioplegia was perfused through the opening of coronary artery. After that, surgeon checked the diseased condition of the aortic root and decided whether to repair the aortic valve, whether to perform Bentall surgery or Wheat surgery. After completing the repair of the aortic root, the four-branch prosthetic graft was anastomosed end-to-end with the proximal aorta, and the proximal end of the innominate artery and the artery between the innominate artery and the left common carotid artery was clamped. After the rectal temperature drops to 28°C, surgeon removed the clamp of the ascending aorta and continued to clamp the innominate artery and the artery between the innominate artery and the left common carotid artery, and then completed the anastomosis of the four-branch prosthetic graft with the distal aorta without circulatory arrest. Subsequently, the left common carotid artery, innominate artery, and left subclavian artery were anastomosed end-to-end with the four-branch prosthetic graft in turn, and then the body temperature was restored and the cardiopulmonary bypass was stopped.

For AAS patients whose lesions only involve the aortic arch but not the ascending aorta, because there was no need to repair the ascending aorta, the procedure did not include cardiopulmonary bypass. The ascending aorta was clamped by the lateral wall clamp, and the Y-shaped artificial aortic vessel was anastomosed end-to-side with the lateral wall of the aorta. Then the innominate artery was cut and clamped, and the stump of the innominate artery was sutured. The distal end of the innominate artery was anastomosed end-to-end with the branch of the Y-shaped prosthetic graft, and the blood supply of the innominate artery was restored after de-airing. Next, using the same method, the left common carotid artery and the left subclavian artery were anastomosed end-to-end with the same branch of the Y-shaped prosthetic graft. In addition, a straight prosthetic graft was wrapped around the ascending aorta and used as a proximal anchoring area during endovascular treatment.

Next, the endovascular treatment part of hybrid surgery was performed. The femoral artery was used as the entry site for endovascular treatment. The stiff guide wire was advanced to the ascending aorta, and the stent graft was placed along the guide wire. The stent graft was released in the four-branch prosthetic graft or the straight prosthetic graft. If the secondary tear was found in the distal aorta or the first stent graft was insufficient to cover the diseased descending aorta, then the second stent graft was used for treatment. After the endovascular treatment was completed, the sternum was sutured and the incision was closed (Figure 1B).

### Follow-Up and Definition

All patients in this study were followed up by telephone or online communication. The follow-up information mainly included the patient's survival status, death time, cause of death, adverse cardiovascular events, neurological function status, and other organ complications. Among them, adverse cardiovascular events were defined as recurrence of AAS or reoperation due to cardiovascular disease.

In this study, emergency surgery referred to surgery performed within 24 h after admission. Permanent neurological complications referred to the obvious abnormal changes found in the brain computed tomography or magnetic resonance imaging or the patient's permanent neurological deficits, mainly including cerebral infarction, cerebral hemorrhage or hemiplegia. Transient neurological complication referred to the brain computed tomography or magnetic resonance imaging without obvious abnormal changes, the patient had transient neurological deficit, and the neurological function has been cured when discharged from the hospital. In the long-term follow-up, neurological complications refer to the symptoms of neurological ischemia or neurological deficits in patients, including dizziness, paraplegia, or inability to walk. New-onset neurological complications refer to patients who did not develop permanent neurological complications during hospitalization, but neurological complications occurred during long-term follow-up.

### Statistical Analysis

The description of the data and basic statistical analysis were performed using R 4.0.4. Continuous variables are expressed as mean ± standard deviation or median (interquartile range). Categorical variables are expressed as frequencies (*n*) with percentages (%). Statistical analysis of continuous variables was performed using Student's *t*-test or Mann-Whitney *U*-test, while categorical variables were analyzed using the chi-square test and Fisher's exact test. Kaplan-Meier test was used to analyze the survival rate of patients. In addition, propensity score matching (PSM) completed using R 4.0.4 was used to obtain patients in the FET group and hybrid surgery group with similar baselines, the variables used included all pre-operative demographic variables, type of AAS, previous non-cardiac surgery, diabetes, emergency surgery, and aortic valve surgery. The matching ratio of the patient was 1:2, and the matching method was nearest neighbor matching with the caliper size set to 0.1 standard deviation. Due to the higher loss of follow-up rate of patients in the FET group, all patients in the FET group who were lost to follow-up were not included in the PSM. In all statistical analyses, *p*-value < 0.05 were considered statistically significant, and all statistical tests used a two-sided test.

## Results

### Baseline Characteristics

The study included a total of 549 patients, including 518 patients underwent TAR with FET and 31 patients underwent hybrid surgery. The basic characteristics and pre-operative data of the patients were listed in Table 1. The basic characteristics of the two groups of patients were similar. However, for patients who were older or had been onset for more than 14 days, surgeons were more inclined to choose hybrid surgery. Therefore, the patients who underwent hybrid surgery were older, and fewer patients had the onset within 14 days. The AAS types of patients with the two surgical methods were also different. All PAU patients were treated with hybrid surgery. In comparison, the proportion of AD patients in the patients with hybrid surgery was relatively small (90.3 vs. 99.0%). This may be related to the slow onset of PAU patients and the longer time to prepare for hybrid surgery. In addition, patients who underwent hybrid surgery had shorter heights and more patients had a history of non-cardiac surgery, which may be related to the patient's old age.

**Table 1:** Demographic and pre-operative data.

	**Overall**			**Propensity score matched**		
	**TAR with FET**	**Hybrid surgery**	***P*-value**	**TAR with FET**	**Hybrid surgery**	***P*-value**
Number of patients (%)	518	31		44	23	
Gender, female (%)	101 (19.5%)	8 (25.8%)	0.446	11 (25.0%)	6 (26.1%)	0.923
Age (years)	48.9 ± 10.8	58.8 ± 11.7	<0.001	55.8 ± 9.6	57.2 ± 12.9	0.656
Information on admission						
Within 14 days after onset (%)	429 (82.8%)	18 (58.1%)	0.001	33 (75.0%)	15 (65.2%)	0.399
Pulse (beats/min)	80.2 ± 12.4	81.1 ± 14.6	0.709	79.3 ± 13.2	83.0 ± 15.8	0.314
Systolic pressure (mmHg)	130.5 (30.0)	130.0 (26.0)	0.448	139.0 (40.0)	130.0 (27.0)	0.254
Diastolic pressure (mmHg)	80.0 (19.0)	75.0 (24.0)	0.149	80.0 (20.0)	75.0 (24.0)	0.135
Height (cm)	171.2 ± 7.8	167.7 ± 7.9	0.016	170.1 ± 7.3	168.4 ± 7.9	0.386
Weight (kg)	75.0 ± 13.2	70.3 ± 11.8	0.056	73.2 ± 12.3	71.7 ± 12.9	0.661
Body mass index (kg/m^2^)	25.5 ± 3.8	24.9 ± 3.4	0.411	25.1 ± 3.1	25.3 ± 3.9	0.854
Type of acute aortic syndrome			<0.001			0.636
Aortic dissection (%)	513 (99.0%)	28 (90.3%)		43 (97.7%)	22 (95.7%)	
Intramural aortic hematoma (%)	5 (1.0%)	1 (3.2%)		1 (2.3%)	1 (4.3%)	
Penetrating atherosclerotic aortic ulcer (%)	0 (0.0%)	2 (6.5%)		0 (0.0%)	0 (0.0%)	
Medical history						
Hypertension (%)	393 (75.9%)	21 (67.7%)	0.279	36 (81.8%)	16 (69.6%)	0.253
Coronary artery disease (%)	32 (6.2%)	4 (12.9%)	0.138	7 (15.9%)	3 (13.0%)	1.000
Diabetes (%)	25 (4.8%)	4 (12.9%)	0.074	2 (4.5%)	1 (4.3%)	1.000
Chronic respiratory disease (%)	13 (2.5%)	1 (3.2%)	0.564	1 (2.3%)	1 (4.3%)	1.000
Renal insufficiency (%)	22 (4.2%)	3 (9.7%)	0.161	7 (15.9%)	3 (13.0%)	1.000
Previous cerebrovascular disease (%)	21 (4.1%)	2 (6.5%)	0.379	3 (6.8%)	0 (0.0%)	0.546
With other chronic diseases (%)	5 (1.0%)	1 (3.2%)	0.297	1 (2.3%)	0 (0.0%)	1.000
Smoking history (%)	212 (40.9%)	16 (51.6%)	0.241	21 (47.7%)	12 (52.2%)	0.730
Previous cardiac surgery (%)	24 (4.6%)	1 (3.2%)	1.000	5 (11.4%)	1 (4.3%)	0.656
Previous non-cardiac surgery (%)	85 (16.4%)	10 (32.3%)	0.023	8 (18.2%)	6 (26.1%)	0.532
Echocardiographic results						
Left ventricular ejection fraction (%)	61.0 (8.0)	62.5 (9.5)	0.076	64.0 (7.8)	63.0 (8.0)	0.634
Pre-operative laboratory examination results						
Absolute value of erythrocyte (10^12^/L)	4.46 ± 1.63	4.15 ± 0.65	0.325	4.32 ± 0.73	4.18 ± 0.69	0.453
Absolute value of leukocyte (10^9^/L)	11.5 ± 5.1	10.1 ± 4.6	0.154	10.5 ± 4.7	10.8 ± 5.0	0.810
Platelet (10^9^/L)	186.6 ± 86.1	213.7 ± 89.3	0.112	178.8 ± 85.1	209.4 ± 76.0	0.174
Hemoglobin (g/L)	133.3 ± 22.3	128.6 ± 19.4	0.282	128.6 ± 21.7	129.5 ± 20.8	0.876
Creatinine (μmol/L)	85.5 (43.6)	82.0 (34.0)	0.806	91.2 (80.7)	92.7 (38.1)	0.817
eGFR (ml/min/1.73 m^2^)	88.5 (42.7)	88.2 (38.9)	0.296	76.7 (57.0)	81.2 (54.8)	0.690
INR	1.09 (0.15)	1.05 (0.19)	0.294	1.07 (0.19)	1.09 (0.20)	0.881
APTT (s)	32.3 (7.3)	29.2 (6.7)	0.013	33.6 (7.0)	30.2 (7.8)	0.093
Albumin (g/mL)	38.7 ± 18.0	40.7 ± 11.0	0.584	37.3 ± 5.8	40.9 ± 10.9	0.192
Fasting blood glucose (mmol/L)	7.30 ± 2.56	6.70 ± 2.06	0.426	6.97 ± 2.59	6.58 ± 2.14	0.674

*eGFR, Estimated Glomerular Filtration Rate; INR, International Normalized Ratio; APTT, activated partial thromboplastin time. The categorical variables in the table are presented by the number of cases (with percentage) and the continuous variables are expressed by the median (with interquartile range) or mean (with standard deviation)*.

PSM was used in the study to eliminate bias caused by different baseline characteristics. A total of 67 patients were identified through PSM, of which 44 patients underwent TAR with FET and 23 patients underwent hybrid surgery. The basic characteristics and pre-operative data of these patients were listed in Table 1. There was no significant difference in the baseline characteristics of patients who underwent the two types of surgery.

### Surgical Data

The surgical data were listed in Table 2. All operations were successfully treated, and no endoleaks were found in the CT examination after the operation, and no caudal migration of the endograft occurred. Compared with patients who underwent hybrid surgery, most patients who underwent TAR with FET were emergency surgery, and there were significantly more patients who had surgery involving the aortic valve or ascending aorta. Among the patients who underwent hybrid surgery, 38.7% of the patients required cardiopulmonary bypass for the operation. Compared with TAR with FET, there was no significant difference in the cardiopulmonary bypass time of these hybrid surgery patients, but the aortic cross-clamp time was significantly reduced, and all hybrid surgery patients did not undergo circulatory arrest. Hybrid surgery reduced intraoperative blood loss but had no significant effect on the operative duration. In the PSM cohort, the difference between the two groups of patients was similar to the overall cohort.

**Table 2:** Intraoperative variables.

	**Overall**			**Propensity score matched**		
	**TAR with FET (*n* = 518)**	**Hybrid surgery (*n* = 31)**	***P*-value**	**TAR with FET (*n* = 44)**	**Hybrid surgery (*n* = 23)**	***P*-value**
Emergency surgery (%)	301 (58.1%)	8 (25.8%)	<0.001	13 (29.5%)	7 (30.4%)	0.940
Concomitant surgery						
Surgery involves aortic valve (%)	269 (51.9%)	3 (9.7%)	<0.001	6 (13.6%)	2 (8.7%)	0.705
Surgery involves mitral valve (%)	16 (3.1%)	0 (0.0%)	1.000	0 (0.0%)	0 (0.0%)	-
Bentall surgery (%)	165 (31.9%)	0 (0.0%)	<0.001	3 (6.8%)	0 (0.0%)	0.546
Cabrol, Wheat or David surgery (%)	73 (14.1%)	1 (3.2%)	0.104	3 (6.8%)	0 (0.0%)	0.546
Ascending aorta replacement surgery (%)	502 (96.9%)	12 (38.7%)	<0.001	43 (97.7%)	8 (34.8%)	<0.001
CABG (%)	31 (6.0%)	1 (3.2%)	1.000	1 (2.3%)	0 (0.0%)	1.000
Surgery with cardiopulmonary bypass (%)	518 (100.0%)	12 (38.7%)	<0.001	44 (100.0%)	8 (34.8%)	<0.001
Cardiopulmonary bypass time (min)	206.0 (71.0)	158.0 (89.3)	0.100	204.5 (67.0)	149.0 (22.0)	0.241
Aortic cross-clamp time (min)	112.0 (53.0)	70.0 (41.0)	0.021	117.0 (54.0)	71.0 (13.0)	0.041
Circulatory arrest time (min)	24.0 (11.0)	0.0 (0.0)	0.001	23.0 (15.0)	0.0 (0.0)	<0.001
Operative duration (hours)	7.16 (2.17)	7.25 (3.25)	0.812	7.04 (2.23)	7.00 (3.75)	0.754
Proximal diameter of stent graft (mm)	-	34.0 (5.0)	-	-	34.0 (3.5)	-
Length of stent graft (mm)	-	200.0 (0.0)	-	-	200.0 (0.0)	-
Intraoperative blood loss (ml)	2350.0 (2440.0)	700.0 (500.0)	<0.001	1860.0 (2150.0)	800.0 (900.0)	<0.001

*CABG, Coronary Artery Bypass Graft. The categorical variables in the table are presented by the number of cases (with percentage) and the continuous variables are expressed by the median (with interquartile range)*.

### Early Outcomes

The short-term prognosis was listed in Table 3. In the overall cohort, there was no significant difference in the 30-day mortality rate and the mortality rate during hospitalization for the two types of surgery, which indicated that the two types of surgery had no effect on the patient's mortality in the short term. Similarly, the two surgical methods had no significant effect on the ventilation time, ICU stays, and hospitalization days. We analyzed the cause of the patient's death during hospitalization. Among patients who underwent TAR with FET, the main causes of death included multiple organ failure (*n* = 21), heart failure (*n* = 11), malignant arrhythmia (*n* = 4), septic shock (*n* = 3), and cerebral hemorrhage (*n* = 2). Among the patients who underwent hybrid surgery, the main causes of death included heart failure (*n* = 2) and cerebral infarction (*n* = 1).

**Table 3:** Short-term Prognosis.

	**Overall**			**Propensity score matched**		
	**TAR with FET (*n* = 518)**	**Hybrid surgery (*n* = 31)**	***P*-value**	**TAR with FET (*n* = 44)**	**Hybrid surgery (*n* = 23)**	***P*-value**
Ventilation time (hours)	39.0 (86.5)	41.0 (104.0)	0.724	70.0 (119.5)	43.0 (105.5)	0.461
ICU stays (days)	3.29 (5.42)	5.00 (8.46)	0.205	4.71 (7.50)	5.00 (8.46)	0.966
Hospitalization days (days)	18.0 (15.0)	21.0 (15.0)	0.181	22.0 (19.8)	21.0 (18.0)	0.737
Post-operative complications (%)	99 (19.1%)	11 (35.5%)	0.027	11 (25.0%)	9 (39.1%)	0.230
Permanent neurological complications (%)	29 (5.6%)	8 (25.8%)	0.001	1 (2.3%)	6 (26.1%)	0.005
Cerebral infarction (%)	12 (2.3%)	7 (22.6%)	<0.001	0 (0.0%)	5 (21.7%)	0.003
Cerebral hemorrhage (%)	9 (1.7%)	1 (3.2%)	0.444	1 (2.3%)	1 (4.3%)	1.000
Hemiplegia (%)	8 (1.5%)	0 (0.0%)	1.000	0 (0.0%)	0 (0.0%)	-
Transient neurological complications (%)	10 (1.9%)	0 (0.0%)	1.000	1 (2.3%)	0 (0.0%)	1.000
Acute renal failure (%)	76 (14.7%)	5 (16.1%)	0.795	8 (18.2%)	4 (17.4%)	1.000
Permanent hemodialysis (%)	8 (1.5%)	1 (3.2%)	0.410	0 (0.0%)	1 (4.3%)	0.343
Acute liver failure (%)	16 (3.1%)	1 (3.2%)	1.000	1 (2.3%)	0 (0.0%)	1.000
Low cardiac output syndrome (%)	21 (4.1%)	0 (0.0%)	0.623	1 (2.3%)	0 (0.0%)	1.000
Pulmonary infection (%)	58 (11.2%)	1 (3.2%)	0.235	4 (9.1%)	1 (4.3%)	0.653
Tracheostomy (%)	18 (3.5%)	1 (3.2%)	1.000	4 (9.1%)	1 (4.3%)	0.653
Reoperation (%)	38 (7.3%)	0 (0.0%)	0.156	6 (13.6%)	0 (0.0%)	0.087
Reoperation for bleeding (%)	22 (4.2%)	0 (0.0%)	0.628	3 (6.8%)	0 (0.0%)	0.546
30-day mortality (%)	55 (10.6%)	3 (9.7%)	1.000	7 (15.9%)	3 (13.0%)	1.000
In-hospital mortality (%)	41 (7.9%)	3 (9.7%)	0.730	7 (15.9%)	3 (13.0%)	1.000

*ICU, Intensive Care Unit. The categorical variables in the table are presented by the number of cases (with percentage) and the continuous variables are expressed by the median (with interquartile range)*.

The analysis of short-term post-operative complications found that hybrid surgery increased the incidence of post-operative complications, especially permanent neurological complications (25.8 vs. 5.6%, 95%CI: 4.7–35.7%, *P* = 0.001). Among them, the incidence of cerebral infarction in hybrid surgery patients increased significantly (22.6 vs. 2.3%, *p* < 0.001). But there is no significant difference between the two surgical methods for other complications. In the PSM cohort, the short-term prognosis results were similar to the overall cohort.

### Mid- and Long-Term Outcomes

As of November 2020, excluding 44 patients who died during hospitalization, we had successfully followed up 388 patients, of which 363 cases (76.1%) underwent TAR with FET and 25 cases (89.3%) underwent hybrid surgery. The average follow-up time was 31.6 months (range between 2 and 82 months). Due to the relatively high rate of loss to follow-up in the FET group, we compared the pre-operative and intraoperative data and short-term prognosis of patients who were lost to follow-up and those who were not lost to follow-up (Supplementary Table 1), and we found that the pre-operative and intraoperative characteristics and short-term prognosis of these two groups of patients were not significantly different.

**Figure 2:**
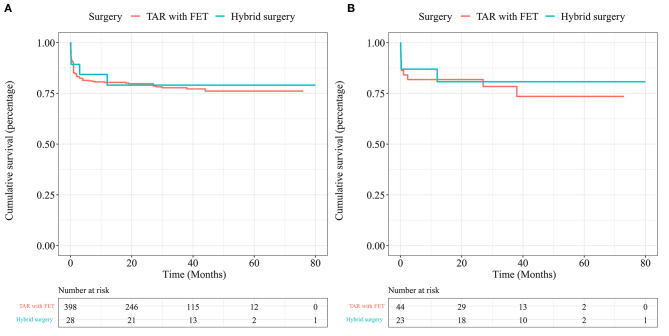
Kaplan-Meier survival curve for TAR with FET and hybrid surgery in the overall cohort **(A)** and in the PSM cohort **(B)**.

In the overall cohort, there was no significant difference in the long-term mortality of the two surgical methods (Log-rank *p* = 0.811). The 1-year survival rate of FET group was 80.4%, the 3-year survival rate was 77.8%, the 1-year survival rate of the hybrid surgery group was 84.3%, and the 3-year survival rate was 79.1% (Figure 2A). In the PSM cohort, there was no significant difference in the long-term mortality of the two surgical methods (Figure 2B).

The follow-up results were listed in Table 4, excluding patients who died during the follow-up. In the overall cohort, patients in the hybrid surgery group had more post-operative complications, especially neurological complications. Compared with patients in the FET group, significantly more patients in the hybrid surgery group had dizziness, inability to walk, and hoarseness. The higher incidence of neurological complications in hybrid surgery patients also increased the rate of rehospitalization. Among the hybrid surgery patients who were re-hospitalized after the operation, 50.0% of the patients were hospitalized for neurological complications. Other reasons for rehospitalization included infection (25.0%) and respiratory failure (25.0%). However, the main reasons for the rehospitalization of TAR with FET patients were coronary heart disease (60.0%) and abdominal aortic aneurysm repair (40.0%). In addition, the proportion of patients in the hybrid surgery group who needed to be hospitalized again increased significantly. At the same time, we analyzed the incidence of new neurological complications. The incidence of new-onset neurological complications in the hybrid surgery group was significantly increased, but most of the new-onset neurological complications were dizziness, and there was no significant difference in the incidence of new-onset paraplegia and inability to walk. In the PSM cohort, the long-term prognosis of the two surgical methods was similar to that of the overall cohort, but there was no significant difference in the incidence of unable to walk, hoarseness, new-onset neurological complications and re-hospitalization between the two groups.

**Table 4:** Mid- and long-term prognosis and follow-up results.

	**Overall**			**Propensity score matched**		
	**TAR with FET (*n* = 311)**	**Hybrid surgery (*n* = 23)**	***P*-value**	**TAR with FET (*n* = 37)**	**Hybrid surgery (*n* = 20)**	***P*-value**
Renal insufficiency (%)	10 (3.2%)	1 (4.3%)	0.549	0 (0.0%)	1 (5.0%)	0.351
Liver insufficiency (%)	3 (1.0%)	1 (4.3%)	0.249	1 (2.7%)	1 (5.0%)	1.000
Neurological complications (%)	53 (17.0%)	12 (52.2%)	<0.001	3 (8.1%)	9 (45.0%)	0.002
Dizziness (%)	28 (9.0%)	8 (34.8%)	0.001	3 (8.1%)	7 (35.0%)	0.024
Paraplegia (%)	8 (2.6%)	1 (4.3%)	0.478	0 (0.0%)	1 (5.0%)	0.351
Unable to walk (%)	9 (2.9%)	4 (17.4%)	0.008	0 (0.0%)	2 (10.0%)	0.119
New-onset neurological complication (%)	49 (15.8%)	8 (34.8%)	0.038	3 (8.1%)	6 (30.0%)	0.054
Dizziness (%)	28 (9.0%)	7 (30.4%)	0.005	3 (8.1%)	6 (30.0%)	0.054
Paraplegia (%)	5 (1.6%)	0 (0.0%)	1.000	0 (0.0%)	0 (0.0%)	-
Unable to walk (%)	4 (1.3%)	1 (4.3%)	0.302	0 (0.0%)	0 (0.0%)	-
Hoarseness (%)	44 (14.1%)	8 (34.8%)	0.015	7 (18.9%)	5 (25.0%)	0.736
Limb ischemia (%)	22 (7.1%)	1 (4.3%)	1.000	2 (5.4%)	1 (5.0%)	1.000
Recurrence of cardiovascular disease (%)	0 (0.0%)	1 (4.3%)	0.070	0 (0.0%)	1 (5.0%)	0.357
Re-hospitalization (%)	5 (1.6%)	4 (17.4%)	0.002	1 (2.7%)	3 (15.0%)	0.119
Reoperation (%)	5 (1.6%)	0 (0.0%)	1.000	1 (2.7%)	0 (0.0%)	1.000

*The categorical variables in the table are presented by the number of cases (with percentage)*.

We used logistic regression analysis and Cox regression analysis to explore whether the two surgical methods are independent risk factors for short-term mortality, long-term mortality, and neurological complications (Table 5). We found that the surgical method was not the independent risk factor for post-operative mortality during hospitalization and long-term mortality. Risk factors for mortality during post-operative hospitalization included coronary heart disease, while protective factors included ascending aortic replacement surgery, higher left ventricular ejection fraction, and higher pre-operative platelets. Risk factors for long-term mortality after surgery included previous cerebrovascular disease, previous cardiac surgery, emergency surgery, and surgery combined with coronary artery bypass graft. However, hybrid surgery was an independent risk factor for permanent post-operative neurological complications (OR = 5.304, 95%CI: 2.120–13.271, *p* < 0.001) and long-term post-operative neurological complications (OR = 5.791, 95%CI: 2.087–16.067, *p* < 0.001). In addition, the risk factors for permanent neurological complications after surgery also included diabetes, and the risk factors for long-term neurological complications after surgery also included coronary heart disease. This indicated that hybrid surgery can lead to an increased risk of short-term and long-term neurological complications.

**Table 5:** Multivariate regression results of post-operative mortality and neurological complications.

**Characteristics**	**B**	***P-*value**	**OR value**	**OR 95%CI**
**Short-term prognosis**				
Permanent neurological complications				
Hybrid surgery	1.668	<0.001	5.304	2.120–13.271
Diabetes	1.145	0.042	3.141	1.043–9.458
Mortality during hospitalization				
Coronary artery disease	1.219	0.042	3.384	1.048–10.930
Ascending aorta replacement surgery	−2.189	0.001	0.112	0.030–0.425
LVEF (%)	−0.052	0.046	0.949	0.902–0.999
Platelet (10^9^/L)	−0.010	0.007	0.990	0.982–0.997
**Long-term prognosis**				
Neurological complications				
Hybrid surgery	1.756	<0.001	5.791	2.087–16.067
Coronary artery disease	1.315	0.044	3.723	1.033–13.416
All-cause mortality	**B**	***P*** **-value**	**HR value**	**HR 95%CI**
Previous cerebrovascular disease	1.574	<0.001	4.825	2.382–9.775
Previous cardiac surgery	0.925	0.024	2.522	1.127–5.644
Emergency surgery	1.016	<0.001	2.761	1.652–4.615
Combined CABG surgery	1.094	<0.001	2.985	1.565–5.692

*LVEF, Left Ventricular Ejection Fraction; CABG, Coronary Artery Bypass Graft; OR, Odds Ratio; HR, Hazard Ratio. The variables considered in the multivariate regression analysis included surgery method, gender, age, onset within 14 days, BMI, hypertension, coronary artery disease, diabetes, chronic respiratory disease, renal insufficiency, previous cerebrovascular disease, smoking history, previous cardiac surgery, LVEF, absolute value of erythrocyte, absolute value of leukocyte, platelets, emergency surgery, ascending aorta replacement surgery, CABG, cardiopulmonary bypass time, aortic cross-clamp time*.

## Discussion

AAS is a high-mortality aortic disease, and its post-operative mortality is about 10–26% (11–13). The treatment of AAS is still a challenge for cardiovascular surgeons (12). Although AAS disease can be treated by a variety of surgical methods, the choice of surgical methods still needs further research (14). Different surgical methods may have differences in the cure effect of the disease, the incidence of complications, and post-operative mortality, which may affect the patient's re-admission, poor quality of life, or death after surgery. The current surgical methods for Type A AAS involving the aortic arch mainly include TAR with FET or elephant trunk and hybrid surgery (8). At present, TAR with FET or hybrid surgery is often used to treat type A AAS in Asia, but there are few studies comparing TAR with FET or hybrid surgery (15–17). Therefore, whether hybrid surgery is more advantageous than FET requires further research.

TAR with FET has been widely used for a long time, and long-term studies in multiple centers have shown that its treatment effect is better, the mortality rate is relatively low, and the prognosis is relatively good (18, 19). FET can maintain distal perfusion by covering the tears of the descending aorta and expanding the true lumen, which can reduce post-operative and long-term aortic events and reduce mortality. In our study, we found that the mortality rate of TAR with FET is similar to the previous report. However, FET requires hypothermic circulatory arrest and complex surgery in the aortic arch, which is generally considered to increase the risk of serious complications after TAR with FET surgery (20, 21).

Hybrid surgery has attracted attention because it does not require hypothermic circulatory arrest and can avoid cardiopulmonary bypass and myocardial ischemia according to the condition (22). In our research, we found that even for patients who require cardiopulmonary bypass for ascending aorta repair, hybrid surgery can still shorten the aortic cross-clamp time to reduce myocardial ischemia, and it also reduces intraoperative blood loss. Because of this advantage, hybrid surgery is usually applied to older patients in the hope of reducing surgical trauma and surgical risk. However, whether hybrid surgery has advantages over TAR with FET in terms of post-operative mortality and complication rate is still uncertain.

In this study, we compared the results of 518 TAR with FET patients and 31 hybrid surgery patients, and used PSM and multivariate regression analysis to control for confounding factors. The post-operative mortality of surgery is an important indicator for evaluating surgical methods. In our study, it was found that the short-term 30-day mortality rate and the long-term 1- and 3-year mortality rates of the two surgical methods were similar. In the recent studies of hybrid surgery, it was found that the in-hospital mortality rate of hybrid surgery for type A aortic dissection is about 6.0–9.2% (23, 24), which is similar to the mortality rate of FET surgery. In addition, in the multivariate regression analysis of this study, the surgical method is not an independent predictor of short-term and long-term post-operative mortality. Therefore, hybrid surgery may have post-operative mortality similar to FET, and it will not increase the patient's risk of death.

Permanent neurological complications after surgery will affect the patient's quality of life and increase the risk of rehospitalization. An observational study comparing open surgery and hybrid surgery conducted by Preventza etale found that the risk of neurological events after hybrid surgery is increased (16). Earlier, Benedetto etale synthesized and analyzed four observational studies (15). The results of their studies suggest that hybrid surgery may increase the incidence of permanent neurological deficits after surgery. However, there was no statistically significant difference in the incidence of neurological events between the two surgical methods in the above research results (*P* > 0.05). Permanent neurological complications after hybrid surgery are considered to be related to aortic atherosclerosis. The surgical operation in the aortic arch and the delivery and release of stents may cause the rupture of atherosclerotic plaques and cause permanent neurological complications (8, 25, 26). In addition, 61.3% of the hybrid surgery patients in this study did not undergo cardiopulmonary bypass during the operation. In these patients, the ascending aorta was clamped by the lateral wall clamp so as to be anastomosed with the prosthetic graft. Side-clamping of the ascending aorta may cause the atherosclerotic plaque of the ascending aorta to rupture and cause neurological complications (27). In this study, we found that whether in the overall cohort or in the PSM cohort, hybrid surgery can increase the occurrence of permanent neurological complications during hospitalization. Hybrid surgery is an independent risk factor for permanent neurological complications (OR = 5.304, *p* < 0.001), and it also increases the occurrence of long-term neurological complications. The incidence of neurological complications reported in this study is higher than that in previous studies. The possible reason is that patients with hybrid surgery have higher diabetes and coronary heart disease, which are all related to atherosclerosis. However, the relationship between permanent neurological complications after hybrid surgery and atherosclerosis still needs further research.

This study has some limitations. First, the inherent flaws of retrospective research are the main limitation of this research. Second, in this study, the number of patients in the hybrid surgery group was small, and a larger amount of data was needed to reduce bias. Third, there is a difference between the baselines of the two groups of patients. Although we use PSM to avoid the impact of the baseline difference, due to the small caliper value set during the PSM process, it is impossible to find a sufficient number of corresponding cases. This may affect the analysis results of the PSM cohort. Fourth, there were fewer patients with a follow-up period of more than 4 years in this study, which may affect the results of survival analysis for more than 4 years. Finally, this multi-center study did not conduct a complete imaging follow-up, so imaging differences between the two groups of patients were not analyzed.

In summary, as a long-term use and approved surgical method, TAR with FET has acceptable short-term and long-term prognosis. Hybrid surgery has the advantages of avoiding hypothermic circulatory arrest, reducing the time of aortic cross-clamp and reducing intraoperative bleeding. Its short-term and long-term mortality are similar to TAR with FET. However, hybrid surgery may increase the risk of permanent neurological complications after surgery. More research is needed to confirm the impact of hybrid surgery on post-operative permanent neurological complications. When choosing hybrid surgery to treat AAS, the risk of post-operative neurological complications must be carefully considered.

## Data Availability Statement

The raw data supporting the conclusions of this article will be made available by the authors, without undue reservation.

## Ethics Statement

The studies involving human participants were reviewed and approved by the ethics committee of Beijing Anzhen Hospital. Written informed consent for participation was not required for this study in accordance with the national legislation and the institutional requirements.

## Author Contributions

MG designed the research. JL, LL, and MW analyzed the data and wrote the paper. HL, LS, YL, RF, ZZ, CZ, and HZ were responsible for data collection. All authors read and approved the final manuscript.

## Conflict of Interest

The authors declare that the research was conducted in the absence of any commercial or financial relationships that could be construed as a potential conflict of interest.

## Publisher's Note

All claims expressed in this article are solely those of the authors and do not necessarily represent those of their affiliated organizations, or those of the publisher, the editors and the reviewers. Any product that may be evaluated in this article, or claim that may be made by its manufacturer, is not guaranteed or endorsed by the publisher.
