# Supplementary material for: Comparison of Prognosis Between Hybrid Debranching Surgery and Total Open Arch Replacement With Frozen Elephant Trunk for Type A Acute Aortic Syndrome Patients
Source: Front Cardiovasc Med. 2021 Jul 27;8:689507. doi: 10.3389/fcvm.2021.689507 (PMC8353071; doi:10.3389/fcvm.2021.689507)
Supplement: Supplementary file 1 [file Table_1.DOCX]

Supplementary Material

# Supplementary Tables

| **Supplementary Table 1 Comparison of Patients Lost to Follow-up and Not Lost to Follow-up** | | | |
| --- | --- | --- | --- |
|  | Not lost to follow-up | Lost to follow-up | P value |
| Number of patients (case) | 363 | 114 |  |
| Gender, female (case) | 68 (18.7%) | 28 (24.6%) | 0.240 |
| Age (years) | 48.6 ± 10.8 | 49.3 ± 10.3 | 0.497 |
| Information on admission |  |  |  |
| Within 14 days after onset (case) | 297 (81.8%) | 96 (84.2%) | 0.559 |
| Pulse (beats/min) | 79.6 ± 12.1 | 81.8 ± 12.2 | 0.090 |
| Systolic pressure (mmHg) | 132.5 (30.0) | 130.0 (32.5) | 0.315 |
| Diastolic pressure (mmHg) | 80.0 (19.0) | 78.5 (20.0) | 0.347 |
| Height (cm) | 171.6 ± 7.4 | 170.1 ± 8.2 | 0.072 |
| Weight (kg) | 74.6 ± 12.6 | 74.5 ± 13.4 | 0.953 |
| Body mass index (kg/m^2^) | 25.3 ± 3.7 | 25.7 ± 3.7 | 0.408 |
| Medical history |  |  |  |
| Hypertension (case) | 277 (76.3%) | 85 (74.6%) | 0.602 |
| Coronary artery disease (case) | 16 (4.4%) | 6 (5.3%) | 0.708 |
| Diabetes (case) | 15 (4.1%) | 6 (5.3%) | 0.603 |
| Chronic respiratory disease (case) | 8 (2.2%) | 3 (2.6%) | 0.731 |
| Renal insufficiency (case) | 13 (3.6%) | 6 (5.3%) | 0.416 |
| Previous cerebrovascular disease (case) | 12 (3.3%) | 5 (4.4%) | 0.569 |
| With other chronic diseases (case) | 5 (1.4%) | 0 (0.0%) | 0.344 |
| Smoking history (case) | 138 (38.0%) | 50 (43.9%) | 0.265 |
| Previous cardiac surgery (case) | 18 (5.0%) | 2 (1.8%) | 0.182 |
| Previous non-cardiac surgery (case) | 57 (15.7%) | 22 (19.3%) | 0.368 |
| Echocardiographic results |  |  |  |
| Left ventricular ejection fraction (%) | 61.0 (7.0) | 63.0 (9.0) | 0.105 |
| Preoperative laboratory examination results |  |  |  |
| Absolute value of erythrocyte (10^12^/L) | 4.50 ± 1.90 | 4.36 ± 0.61 | 0.449 |
| Absolute value of leukocyte (10^9^/L) | 10.9 ± 4.1 | 12.6 ± 7.1 | 0.021 |
| Platelet (10^9^/L) | 188.6 ± 91.3 | 192.7 ± 75.2 | 0.669 |
| Hemoglobin (g/L) | 133.5 ± 23.8 | 132.1 ± 16.8 | 0.496 |
| Creatinine (μmol/L) | 83.8 (40.5) | 85.6 (45.6) | 0.703 |
| eGFR (ml/min/1.73m^2^) | 89.0 (42.2) | 90.3 (38.9) | 0.537 |
| INR | 1.09 (0.15) | 1.08 (0.15) | 0.534 |
| APTT (s) | 32.3 (7.6) | 31.7 (7.0) | 0.979 |
| Albumin (g/mL) | 39.1 ± 21.2 | 37.6 ± 4.9 | 0.480 |
| Fasting blood glucose (mmol/L) | 7.19 ± 2.50 | 7.66 ± 2.60 | 0.089 |
| Emergency surgery (case) | 200 (55.1%) | 71 (62.3%) | 0.177 |
| Concomitant surgery |  |  |  |
| Surgery involves aortic valve (case) | 193 (53.2%) | 56 (49.1%) | 0.451 |
| Surgery involves mitral valve (case) | 9 (2.5%) | 5 (4.4%) | 0.338 |
| Bentall surgery (case) | 117 (32.2%) | 33 (28.9%) | 0.510 |
| Cabrol, Wheat or David surgery (case) | 53 (14.6%) | 17 (14.9%) | 0.935 |
| Ascending aorta replacement surgery (case) | 353 (97.2%) | 112 (98.2%) | 0.740 |
| CABG (case) | 18 (5.0%) | 5 (4.4%) | 0.803 |
| Cardiopulmonary bypass time (min) | 204.0 (68.3) | 212.5 (77.3) | 0.509 |
| Aortic cross-clamp time (min) | 111.0 (55.5) | 111.0 (54.0) | 0.800 |
| Operative duration (hours) | 7.08 (2.08) | 7.00 (2.00) | 0.161 |
| Intraoperative blood loss (ml) | 2300.0 (2367.5) | 2200.0 (1900.0) | 0.845 |
| Ventilation time (hours) | 39.0 (88.0) | 34.0 (77.8) | 0.724 |
| ICU stays (days) | 3.42 (5.40) | 2.94 (5.32) | 0.840 |
| Hospitalization days (days) | 20.0 (15.0) | 16.0 (14.0) | 0.019 |
| Postoperative complications (case) | 52 (14.3%) | 17 (14.9%) | 0.876 |
| Permanent neurological complications (case) | 16 (4.4%) | 6 (5.3%) | 0.704 |
| Cerebral infarction (case) | 5 (1.4%) | 4 (3.5%) | 0.227 |
| Cerebral hemorrhage (case) | 4 (1.1%) | 1 (0.9%) | 1.000 |
| Hemiplegia (case) | 7 (1.9%) | 1 (0.9%) | 0.687 |
| Transient neurological complications (case) | 9 (2.5%) | 1 (0.9%) | 0.464 |
| Acute renal failure (case) | 40 (11.0%) | 19 (16.7%) | 0.110 |
| Acute liver failure (case) | 6 (1.7%) | 4 (3.5%) | 0.260 |
| Low cardiac output syndrome (case) | 2 (0.6%) | 3 (2.6%) | 0.091 |
| Pulmonary infection (case) | 40 (11.0%) | 17 (14.9%) | 0.264 |
| Reoperation (case) | 26 (7.2%) | 4 (3.5%) | 0.161 |
| Abbreviations: eGFR, Estimated Glomerular Filtration Rate; INR, International Normalized Ratio; APTT, activated partial thromboplastin time; CABG, Coronary Artery Bypass Graft; ICU, Intensive Care Unit. NOTE. The categorical variables in the table are presented by the number of cases (with percentage) and the continuous variables are expressed by the median (with interquartile range) or mean (with standard deviation). | | | |
